# How I diagnose and treat patients in the pre-fibrotic phase of primary myelofibrosis (pre-PMF) - practical approaches of a German expert panel discussion in 2024

**DOI:** 10.1007/s00277-025-06191-7

**Published:** 2025-01-31

**Authors:** Martin Griesshammer, Haifa Kathrin Al-Ali, Jan-Niklas Eckardt, Michael Fiegl, Joachim Göthert, Kathleen Jentsch-Ullrich, Steffen Koschmieder, Hans Michael Kvasnicka, Andreas Reiter, Burkhard Schmidt, Florian H. Heidel

**Affiliations:** 1 Oncology, Haemostaseology and Palliative Care, Johannes Wesling Medical Center Minden, University Clinic for Hematology, University of Bochum, Bochum, Germany; 2 Krukenberg Cancer Center, University Hospital of Halle, Halle, Germany; 3 Department of Internal Medicine I, University Hospital Dresden, Dresden, Germany; 4 Else-Kröner-Fresenius- Center for Digital Health, Dresden University of Technology, Dresden, Germany; 5 Hämatologie Onkologie Germering, Germering, Germany; 6 Department of Hematology and Stem Cell Transplantation, University Hospital Essen, Essen, Germany; 7 Gemeinschaftspraxis Hämatologie und Onkologie, Magdeburg, Germany; 8 Department of Hematology, Oncology, Hemostaseology, and Stem Cell Transplantation, Faculty of Medicine, RWTH Aachen University, Aachen, Germany; 9 Center for Integrated Oncology Aachen Bonn Cologne Düsseldorf (CIO ABCD), Düsseldorf, Germany; 10 Fakultät für Gesundheit (Department für Humanmedizin), Pathologie, Universität Witten/Herdecke, Herdecke, Germany; 11 Department of Hematology and Oncology, University Hospital Mannheim, Mannheim, Germany; 12 Hämatologie und Onkologie München Pasing MVZ GmbH, München, Germany; 13 Hematology, Hemostasis, Oncology and Stem Cell Transplantation, Hannover Medical School (MHH), Hannover, Germany; 14 Cellular Therapy Center (CTC), Hannover Medical School (MHH), Hannover, Germany; 15 Leibniz Institute on Aging, Fritz-Lipmann-Institute, Jena, Germany; 16 Hematology, Hemostasis, Oncology and Stem Cell Transplantation, Hannover Medical School (MHH), Carl-Neuberg-Str. 1, D-30625 Hannover, Germany

**Keywords:** Pre-PMF, Prefibrotic myelofibrosis, MPN, Myeloproliferative neoplasm, Targeted therapy

## Abstract

The prefibrotic phase of primary myelofibrosis (pre-PMF) represents a distinct subentity within the spectrum of myeloproliferative neoplasms (MPNs), recognized by the World Health Organization (WHO) and the International Consensus Classification (ICC). Pre-PMF is characterized by unique morphological, clinical, and molecular features, distinguishing it from essential thrombocythemia (ET) and overt myelofibrosis (overt-PMF). The diagnostic process for pre-PMF relies on bone marrow histology, identification of molecular mutations and exclusion of other myeloid neoplasms. Misclassification remains a significant challenge due to overlapping phenotypes and the heterogeneity of clinical presentations, which range from asymptomatic cases to severe cytopenias and a high thrombotic risk. Management strategies for pre-PMF focus on mitigating symptom burden, reducing thromboembolic events, and preventing disease progression. Low-risk patients often benefit from observational approaches or low-dose aspirin, while cytoreductive therapies, such as hydroxyurea or interferon-alpha, are utilized in symptomatic or high-risk cases. JAK inhibitors like ruxolitinib have shown promise in addressing splenomegaly and systemic symptoms, although their role in pre-PMF requires further investigation. Advances in artificial intelligence are enhancing diagnostic precision by refining bone marrow histopathological analysis, paving the way for more accurate disease classification and tailored therapeutic strategies. This position paper integrates insights from a German expert panel discussion, underscoring the need for interdisciplinary collaboration, adherence to updated WHO/ICC diagnostic criteria, and personalized treatment approaches. By addressing diagnostic challenges and therapeutic nuances, it seeks to improve outcomes and quality of life for patients navigating the complexities of pre-PMF.

## Introduction

The prefibrotic phase of primary myelofibrosis (pre-PMF) is recognized as a separate subentity within the category of Myeloproliferative Neoplasms (MPN), apart from Essential Thrombocythemia (ET), early phases of Polycythemia vera (PV) and the overt or fibrotic phase of PMF, with specific criteria outlined by the World Health Organization (WHO) and International Consensus Classification (ICC) [1, 2]. Misdiagnosis is common due to variability in bone marrow (BM) morphology and lack of clinical and molecular data integration [3]. The clinical presentation of pre-PMF is diverse, leading to heterogeneous management strategies. Clinical challenges and complications include disease-associated symptoms such as fatigue and night sweats, thrombosis, hemorrhage, cytopenias, and - less frequent - progression to overt PMF or blast-phase MPN (BP-MPN) / secondary acute myeloid leukemia (AML). Accurate diagnosis requires integration of clinical, laboratory, genetic and pathological features. As misclassification or misdiagnosis is frequent, the need for multidisciplinary approaches and strong adherence to WHO/ICC criteria needs to be emphasized. Prognostic tools specifically for pre-PMF have not been developed, however, scoring systems for the overt phase can be adapted. The condition is associated with a higher thrombotic risk than ET and can progress to more severe disease phases in individual patients [4]. Management strategies include careful monitoring, cytoreductive therapies, symptomatic treatment approaches and consideration of allogeneic hematopoietic stem cell transplantation (allo-HSCT) in cases with clinical risk factors and genetic/molecular high-risk factors. Advances in genetic profiling can prospectively improve diagnostic accuracy and prognostication and help to differentiate pre-PMF from other MPN subtypes to guide treatment decisions. Recent advances in artificial intelligence (AI) and deep learning (DL) have significantly enhanced the analysis of BM histology [5–7]. AI techniques, including neural networks and support vector machines, are increasingly applied to tasks such as cell detection, recognition, and classification, offering robust image characterization and automatic feature learning capabilities. AI supports the diagnostic process by automating various stages of BM cell morphology analysis, including detection, segmentation, identification, classification, enumeration, and diagnosis. End-to-end deep learning systems have demonstrated high accuracy in detecting and classifying BM cells, facilitating rapid and precise diagnostics. The digitalization of histological slides further enables AI applications in pathology, promising to detect subtle phenotypic changes and improve diagnostic objectivity.

### Diagnostic criteria and terminology

The 2016 World Health Organization (WHO) classification introduced pre-PMF as a distinct subentity within the category of myeloproliferative neoplasms (MPN). This reclassification has posed several challenges in accurately diagnosing and differentiating pre-PMF from other MPNs, particularly essential thrombocythemia (ET) and overt PMF, to a lesser extent also polycythemia vera (PV). The application of the 2016 WHO classification criteria for pre-PMF presents several challenges, primarily due to overlapping clinical, hematological, and molecular features between the various subentities. Accurate diagnosis requires a comprehensive approach integrating morphologic, immunophenotypic, genetic, and cytogenetic data. Continued refinement of diagnostic criteria and increased awareness of the distinct features of pre-PMF are essential for improving diagnostic accuracy and patient outcomes. The 2016 and 2022 WHO criteria as well as the International Consensus Criteria (ICC) for pre-PMF are similar and show only minor differences as follows:

### WHO 2016 diagnostic criteria for pre-fibrotic myelofibrosis (Pre-PMF) [8]

#### Major criteria

1. **Megakaryocyte Proliferation and Atypia**:

- Presence of megakaryocyte proliferation and atypia without reticulin fibrosis grade > 1.
- Age-adjusted increase in BM cellularity with granulocytic proliferation and often decreased erythropoiesis.

2. **Exclusion of Other Myeloid Malignancies:**

- Does not meet the WHO criteria for BCR-ABL1 + chronic myeloid leukemia, polycythemia vera, essential thrombocythemia, myelodysplastic syndromes, or other myeloid neoplasms.

3. **Presence of JAK2, CALR, or MPL Mutation:**

- In the absence of these mutations, the presence of another clonal marker, or no evidence of reactive marrow fibrosis.

#### Minor criteria (at least one must be present)

1. Anemia not attributed to a comorbid condition.
2. Leukocytosis ≥ 11 × 10^9/L.
3. Palpable splenomegaly.
4. Serum lactate dehydrogenase (LDH) level above the upper normal limit.

### WHO 2022 diagnostic criteria for pre-fibrotic myelofibrosis (Pre-PMF) [2]

#### Major criteria

1. **Megakaryocyte Proliferation and Atypia:**

- Presence of megakaryocyte proliferation and atypia without significant reticulin fibrosis (reticulin fibrosis grade 0–1).
- Age-adjusted increase in BM cellularity with granulocytic proliferation and often decreased erythropoiesis.

2. **Exclusion of Other Myeloid Malignancies:**

- Excludes diagnoses such as BCR-ABL1 + chronic myeloid leukemia, polycythemia vera, essential thrombocythemia, myelodysplastic syndromes, and other myeloid neoplasms.

3. **Clonal Marker Evidence:**

- Presence of JAK2, CALR, or MPL mutation, or in the absence of these mutations, another clonal marker indicating a myeloid neoplasm, or absence of reactive BM fibrosis.

### Minor criteria (at least one must be present)

1. Anemia not attributable to a comorbid condition.
2. Leukocytosis ≥ 11 × 10^9/L.
3. Palpable splenomegaly.
4. Serum lactate dehydrogenase (LDH) level above the normal reference range.

Both editions highlight the importance of a comprehensive diagnostic approach that includes morphological evaluation of BM histology, exclusion of other conditions, and the presence of specific genetic mutations. The criteria remain largely consistent, emphasizing the unique characteristics of pre-PMF and its distinction from related MPN.

### ICC 2022 diagnostic criteria for pre-fibrotic myelofibrosis (Pre-PMF) [1]

#### Major criteria

1. **BM biopsy** showing megakaryocytic proliferation and atypia (a), BM fibrosis grade < 2, increased age-adjusted BM cellularity, granulocytic proliferation, and (often) decreased erythropoiesis.
2. **Mutational Status**: JAK2, CALR, or MPL mutation (b) or presence of another clonal marker (c) or absence of reactive BM reticulin fibrosis (d).
3. **Diagnostic criteria for BCR::ABL1-positive chronic myeloid leukemia**,** polycythemia vera**,** essential thrombocythemia**,** myelodysplastic syndromes**,** or other myeloid neoplasms are not met**.

#### Minor criteria

1. Anemia not attributed to a comorbid condition.
2. Leukocytosis ≥ 11 × 10e9/L.
3. Palpable splenomegaly.
4. Lactate dehydrogenase level above the reference range.

The diagnosis of pre-PMF requires all 3 major criteria and at least 1 minor criterion confirmed in 2 consecutive determinations.

- Morphology of megakaryocytes in pre-PMF and overt PMF usually demonstrates a higher degree of megakaryocytic atypia than in any other MPN-subtype; distinctive features of megakaryocytes include small to giant megakaryocytes with a prevalence of severe maturation defects (cloud-like, hypolobulated and hyperchromatic nuclei) and presence of abnormal large dense clusters (mostly > 6 megakaryocytes lying strictly adjacent).
- It is recommended to use highly sensitive assays for JAK2 V617F (sensitivity level < 1%) and CALR and MPL (sensitivity level 1–3%) - in negative cases, consider searching for non-canonical JAK2 and MPL mutations.
- Assessed by cytogenetics or sensitive NGS techniques; detection of mutations associated with myeloid neoplasms (e.g. ASXL1, EZH2, IDH1, IDH2, SF3B1, SRSF2, and TET2 mutations) supports the clonal nature of the disease.
- Minimal reticulin fibrosis (grade 1) secondary to infection, autoimmune disorder or other chronic inflammatory conditions, hairy cell leukemia or another lymphoid neoplasm, metastatic malignancy, or toxic (chronic) myelopathies.

### Review and discussion-based recommendations for the treatment of patients in pre-fibrotic phase of PMF

1. How do we define and diagnose pre-PMF? What are the clinical characteristics of pre-PMF and how do they separate from other subentities of MPN?

Pre-PMF is a subentity within the category of MPNs which is characterized by an increased cellularity with rather decreased erythropoiesis, a dysplastic and clustered megakaryopoiesis without or with minimal myelofibrosis (i.e. BM fibrosis grade 0 or 1 on a scale of 0–3). Diagnosing pre-PMF should always involve a combination of clinical evaluation, laboratory tests, genetic/molecular analysis and histopathological examination of BM samples and can only be successful when all aspects are integrated. There is consensus that expert evaluation (reference pathology) may prevent misdiagnosis and improve the diagnostic assessment of all MPN subtypes (specifically regarding the pre-PMF). Laboratory findings often include elevated white blood cell, platelet counts and LDH. The diagnosis is primarily confirmed through a BM biopsy, which reveals characteristic features such as hypercellularity, megakaryocyte proliferation with atypia, and reticulin fibrosis grade 0–1.

Clinically, patients frequently present with symptoms such as fatigue, night sweats, bone pain and others as well as splenomegaly and mild anemia [3]. Structured assessment of symptoms using validated scales (e.g. MPN10 or MPN-SAF) is clearly recommended, as physician-based assessment may underestimate the complexity of symptoms complex [9, 10].

Genetic testing is crucial for identifying MPN driver mutations in pre-PMF, including JAK2, CALR, and MPL mutations. Moreover, additional somatic mutations including mutations of the high-risk spectrum are relevant [11, 12]. These mutations may help to differentiate pre-PMF from other MPNs like ET. According to the World Health Organization (WHO) 2016 and 2022 and ICC 2022 criteria, the diagnosis of pre-PMF requires the presence of these BM abnormalities along with the exclusion of other hematological neoplasms.

### Relevant aspects of the panel discussion

- It is problematic to diagnose a patient with elevated LDH and leukocytosis with ET in case there is no explanation for these two findings such as infection, hemolysis or smoking. The frequently hypercellular BM in these patients does not align with “classic” ET. However, the hyperplastic BM could also be due to a concurrent inflammatory condition. Without knowledge of the minor criteria, a pathologist cannot diagnose pre-PMF but can only consider this diagnosis depending on the minor criteria.
- Presentation of the clinical and genetic characteristics of WHO-defined pre-PMF and ET are important (WBC, LDH, palpable splenomegaly, degree of fibrosis). Communication between the clinician and the pathologist is essential for an accurate diagnosis. The updated WHO criteria for pre-PMF [13] should be taken into consideration. Both ICC and WHO propose similar criteria: anemia, leukocytosis, palpable splenomegaly, LDH, major and minor criteria. Some patients will only show one minor criterion. Only few patients do not show any minor criteria and therefore fall under unclassified MPNs (thus not pre-PMF!). Without a minor criterion, the diagnosis of pre-PMF according to the WHO classification is not possible. It is important not to diagnose pre-PMF too hastily, so the patient does not receive this disease “label”. Rather, an “early form of MPN” could be diagnosed, with comments suggesting the possibility of pre-PMF or ET, indicating differential diagnoses based on clinical findings and recommendation of timely re-assessment. LDH is one of the more important parameters of pre-PMF.
- Morphologically, pre-PMF is a different disease entity compared to the other MPN subentities. A hypercellular BM does not fit with ET, only with PV. The majority of megakaryocytes must be atypical for the diagnosis of pre-PMF, for example, hyperchromasia, condensed nuclei, or myelodysplastic syndrome (MDS)-like megakaryocytes, and histo-topography, such as the tendency of megakaryocytes to group. They can be found in loose groups, but clustering is more likely in pre-PMF. Clusters of 6 or more megakaryocytes are considered atypical and are more indicative of pre-PMF [1]. Clusters of fewer than 6 megakaryocytes can also occur in ET. A hypercellular BM with small clusters of megakaryocytes cannot be ET. Similarly, a BM in which megakaryocytes encircle adipocytes does not fit with ET. Erythropoiesis in pre-PMF is typically dysplastic and left-shifted, often with increased numbers of immature erythroid precursors, reflecting ineffective hematopoiesis. In contrast, PV shows hyperplastic erythropoiesis, characterized by an abundance of mature erythroid precursors.
- Frequently, patients with CMML (MDS/MPN overlap) have also been diagnosed as pre-PMF, especially if they have JAK mutations, as they are seen as MPN due to the presence of atypical megakaryocytes. In patients with monocytosis, the molecular findings must be integrated into the diagnosis (particularly TET2, ASXL1, SRSF2, SF3B1), as monocytosis is more indicative of an MDS/MPN rather than pre-PMF [14–16]. Monocytosis is a prognostic marker in PMF, not a diagnostic criterion.
- Not every case of pre-PMF progresses to overt PMF. Pre-PMF is a distinct MPN subgroup that is clinically and genetically different from ET and overt PMF [17]. Many patients with pre-PMF do progress to overt PMF, but the timing of progression to higher grade fibrosis is uncertain; of note, there are no bona fide biomarker established. It may take years (sometimes 10–20 years) before progression of fibrosis occurs. The diagnosis of pre-PMF is therefore particularly relevant for younger patients to ensure the transformation point is not missed especially regarding the probability of transition from pre-PMF to overt PMF, blast phase, and death [18].

2) Are ET and pre-PMF clearly distinguishable entities or rather part of a continuous spectrum?

A study highlights the critical role of accurate morphologic diagnosis in distinguishing between essential thrombocythemia (ET) and pre-PMF, which significantly impacts patient outcomes [19].

Misdiagnosis of overt primary myelofibrosis (PMF) frequently arises from the misinterpretation of fibrosis grades, as cases with bone marrow fibrosis grade 1, which do not meet the criteria for overt PMF, are often erroneously classified as such on pathology reports. This diagnostic error can lead to inappropriate prognostication and treatment strategies, emphasizing the necessity for standardized and precise histological assessments. Additionally, “masked polycythemia vera” (PV), a variant with subtle erythrocytosis or suppressed hemoglobin levels due to plasma volume expansion, is frequently misinterpreted as essential thrombocythemia (ET) or pre-PMF, further complicating accurate disease classification and necessitating integration of clinical, histopathological, and molecular data for proper diagnosis. Many cases initially diagnosed as ET were reclassified as pre-PMF upon detailed histopathological review. This misclassification has substantial implications for prognosis and treatment strategies. Patients with reclassified pre-PMF had worse overall survival compared to those with “true” ET. The study reported that survival in true ET was comparable to the general population, while pre-PMF had a significantly higher risk of mortality. Here, 16% of patients were reclassified as pre-PMF when strict 2016 WHO criteria were applied. Moreover, the two entities (ET compared to pre-PMF) showed differences regarding outcome: overall survival (OS) of 89% versus 76%; progression to AML (0.7% versus 5.8%) and progression to the fibrotic phase of myelofibrosis (0.8% versus 12.3%), respectively. Likewise, diagnostic laboratory findings and clinical characteristics showed differences between ET when compared to pre-PMF: LDH levels (296 versus 429 mU/mL), palpable splenomegaly (16% versus 23%) and presence of fibrosis grade 1 (3% versus 24%). Based on these findings, the lack of accuracy in diagnosing ET versus pre-PMF in real-world settings has been most recently compiled in a chart review on 960 MPN patients [3]: while they met at least one minor WHO-criterion for primary myelofibrosis, 39.8% of those diagnosed with ET did not have histopathological BM testing at diagnosis. 63.4% of patients who were classified as having MF (including pre-MF), however, did not obtain an early prognostic risk assessment. More than 50% patients classified as myelofibrosis showed characteristics consistent with the pre-fibrotic phase, which was emphasized by the frequent use of cytoreductive therapy, which is more frequently indicated and used in the early, proliferative phases of MF. The study found that physicians tended to adopt a more aggressive treatment approach for pre-MF due to its higher risk of progression to overt MF and potential transformation into blast phase/secondary acute myeloid leukemia (AML). In contrast, ET patients, who generally have a more indolent disease course, were managed with less intensive therapies. Treatment patterns varied considerably between patients diagnosed with ET and those with pre- MF. Patients with pre-PMF were more likely to receive cytoreductive therapy, such as hydroxyurea, and required more intensive monitoring compared to those with true ET.

Regarding the thromboembolic risk assessment, the IPSET score, initially developed for ET, has been validated for predicting thrombosis in patients with pre-PMF [20]. Studies have shown that the risk of overall thrombosis predicted by the IPSET score corresponds to 0.67%, 2.05%, and 2.95% patients/year in the low-, intermediate-, and high-risk categories, respectively. The IPSET score has been found to be superior to both the conventional 2-tiered score and the revised IPSET in cohorts of pre-MF patients. Therefore, individualized management aimed at reducing the increased risk of major cardiovascular events can be achieved. Further refinement of the IPSET score in pre-PMF might be pursued by additional, prospective studies evaluating the inclusion of leukocytosis and/or adverse mutational profiles as novel variables.

Overall, while the driver mutation profiles in ET and pre-MF are broadly comparable, pre-PMF tends to have a slightly higher prevalence of JAK2 (50–60% versus 60–70%) mutations [21]. High-Risk Mutations (HMRs) are relatively rare in ET compared to pre-MF. When present, they can indicate a higher risk of disease progression and a potentially poorer prognosis. The lower incidence of HMRs in ET corresponds with the generally more indolent nature of the disease. In pre-PMF, the presence of mutations in ASXL1, SRSF2, EZH2, IDH1/2, and TP53 is significantly higher and is associated with a worse prognosis, including a higher likelihood of progression to overt myelofibrosis, AML, and reduced overall survival [11, 19, 22–24]. Several scoring systems are available for assessing the risk of progression in pre-PMF, each with its strengths and limitations. Modern scoring systems like GIPSS and MIPSSv2 as well as extensive analyses of the MPN genomic landscape [11] emphasize the importance of genetic mutations and karyotype information. These systems provide a more precise risk assessment by incorporating molecular data, which is crucial for understanding disease progression [25]. This is of specific importance as patients diagnosed with pre-PMF categorized as low- or intermediate risk by clinically inspired scoring systems may show a molecular high-risk status [12, 26]. Approximately 40% of IPSS/DIPSS-Plus lower-risk patients are considered high molecular risk [27].

### Relevant aspects from the panel discussion

- Symptoms and spleen size are a continuum, as is the case between ET and pre-PMF. Over 95% of patients report on symptoms, when structurally assessed using questionnaires. Patients will report their symptoms if given sufficient time and structured assessment forms. The vast majority of patients experience fatigue, but to very different degrees of severity. Structured documentation of symptoms is therefore important: significantly more patients report symptoms than is perceived, but this goes unnoticed if they are not documented on a regular basis and in a structured manner (using validated scales).
- Diagnosis of an MPN subtype cannot be solely deduced from the histopathologically assessed degree of fibrosis. The degree of fibrosis is just one of the parameters, and classification is the best possible approximation of clinical problems. ET and pre-PMF are certainly biologically closely related diseases, but, at the moment, they cannot be better defined. Individual parameters may overlap, but the overall pattern does not. Pre-PMF may sometimes be separated from ET only after a considerable amount of time of disease course. Eventually, genetic parameters may help to explain morphologic findings and clinical phenotype. Currently, categorization as proposed by the WHO/ICC is necessary to guide treatment strategies, however, in the future molecular and clinical biomarkers with predictive value may facilitate phenotype agnostic treatment approaches.
- There is no difference in the treatment goals of ET or pre-PMF purely based on the classification of the disease. If the patient has a high risk for thromboembolic complications or significant symptom burden, there is an indication for cytoreductive or symptom-oriented therapy, similar to what is recommended for high-risk ET. Treatment should be focused on clinical needs and risk factors. Labels of the respective drugs and guidelines allow for treatment within the scope of the approval and beyond. However, most approvals were granted before the re-classification of PMF into prefibrotic and overt phases. Of note, certain patient subgroups may not have been represented sufficiently in the relevant trials at that time.
- MPN show commonalities and unifying mechanisms at the level of the genomic landscape that have similar functional consequences [28]. These overlaps in pathophysiology across this disease spectrum provide common targets for therapeutic intervention and future development of personalized treatments.
- It was demonstrated that 16% (180/1071) of patients initially diagnosed with essential thrombocythemia (ET) could be reclassified as having pre-PMF upon detailed clinical and histopathological evaluation. This reclassification highlights the critical role of accurate differentiation between ET and pre-PMF, given the distinct clinical course and prognostic implications associated with these entities. The findings underscore the need for meticulous diagnostic criteria, including bone marrow morphology and minor clinical features, to refine disease classification and guide appropriate management strategies [19].
- Likewise, recent data revealed that 42% (28/661) of patients initially diagnosed with overt myelofibrosis (MF) could be reclassified as having pre-PMF based on updated WHO diagnostic criteria. This reclassification underscores the overlap in clinical and morphological features between these entities, particularly in early disease stages, and highlights the importance of bone marrow histopathology for precise disease characterization. The findings emphasize the need for accurate diagnostic frameworks to distinguish pre-PMF from overt MF, as they carry distinct prognostic trajectories and therapeutic implications, ultimately impacting patient outcomes [26]. Notably, there is low intra- and interprofessional reproducibility (53–88%).
- JAK2V617F variant allele frequency (VAF) serves as a critical discriminator between essential thrombocythemia (ET) and pre-PMF. In ET, median JAK2 VAFs tend to be lower (around 25%) compared to pre-PMF, where the median VAF is significantly higher (approximately 46%). A JAK2 VAF exceeding 50–60% is concerning at diagnosis for ET, often signaling the need to reconsider the classification towards pre-PMF. For intermediate VAFs (10–20%), additional parameters such as bone marrow histology and the presence of co-mutations, including CALR or MPL, must be integrated into the diagnostic framework to ensure accurate classification and prognostication [29].
- Cytopenic pre-PMF represents a distinct clinical phenotype affecting one or more hematopoietic lineages, including anemia, thrombocytopenia, and leukopenia. Patients with cytopenic pre-PMF exhibit higher frequencies of adverse clinical features such as elevated peripheral blood blasts, splenomegaly, and constitutional symptoms, alongside enriched molecular abnormalities, including high-risk mutations in ASXL1, U2AF1, and other high molecular risk genes. This phenotype is associated with significantly shorter overall survival compared to the proliferative pre-PMF subgroup, reflecting its adverse prognostic implications. Moreover, cytopenic pre-PMF patients have a substantially higher risk of progression to overt fibrotic myelofibrosis, particularly in the presence of anemia and thrombocytopenia. These findings underscore the need for early identification and tailored management strategies to address the poor outcomes associated with the cytopenic phenotype [30].
- Recent data highlighted the significant therapeutic potential of early ruxolitinib (RUX) initiation in pre-PMF, showing clinical benefits irrespective of fibrosis grade. Patients with low-grade fibrosis (LGF) exhibited superior spleen responses, overall survival, and progression-free survival compared to those with high-grade fibrosis (HGF). Importantly, early initiation of RUX, within two years of diagnosis, was associated with markedly improved outcomes in both LGF and HGF patients, emphasizing the progressive nature of the disease and the need for timely intervention. The findings also underscore the manageable safety profile of RUX, even in patients with advanced disease stages. This reinforces the critical role of accurate staging and early therapeutic intervention in optimizing patient outcomes in pre-PMF [31].
- Significant differences in survival and disease characteristics between pre-PMF and overt PMF have been reported, using the 2016 WHO diagnostic criteria [26]. Median overall survival (OS) was notably longer in pre-PMF (14.7 years) compared to overt PMF (7.2 years), with similar trends observed for leukemia-free survival (LFS). Prognostic mutations such as ASXL1 and EZH2 were more frequently observed in overt PMF and were associated with worse outcomes, underscoring the relevance of molecular profiling in risk stratification. Additionally, diagnostic scoring systems like DIPSS and IPSS effectively differentiated risk categories but may require recalibration for the pre-PMF subgroup. These findings emphasize the continuum between pre-PMF and overt PMF and the critical importance of integrating phenotypic and genotypic data for accurate diagnosis and prognosis. The study also noted that the JAK2V617F allelic burden (or variant allele frequency, VAF) is significantly higher in pre-PMF compared to essential thrombocythemia (ET), reflecting the more advanced myeloproliferative phenotype of pre-PMF. This distinction further aids in the differential diagnosis and highlights the progression of clonal hematopoiesis from ET to pre-PMF.

3) What are therapeutic options and how does JAK-inhibitor therapy rank among therapies of pre-PMF? How do I treat pre-PMF?

For low-risk pre-PMF patients (e.g. according to DIPSS- or MIPSS70-scoring) with low symptom burden, observation without active treatment is often recommended. This approach is based on the relatively favorable prognosis and the potential risks associated with unnecessary treatment. Low-dose aspirin may be prescribed to reduce the risk of thrombotic events. This is especially relevant for patients with a history of arterial or venous thrombosis. Hydroxyurea is commonly used as a first-line cytoreductive therapy in patients with thrombocytosis or leukocytosis. In the German MPN guidelines, anagrelide is currently recommended as 1st or 2nd line option in ET [32] and in clinical practice it is also used in patients with pre-PMF and thrombocytosis [3]. However, data on anagrelide-induced marrow fibrosis [28] or at least inability of anagrelide to prevent progression of BM fibrosis as well as frequent cardiac side effects have raised concerns regarding its use especially in younger patients. Interferon alfa is an alternative to hydroxyurea, particularly for younger patients with a long life-expectancy. It is used to control myeloproliferation and has shown efficacy in controlling disease progression in MPN [33]. Currently, Ropeginterferon-alpha is evaluated in clinical phase III trials in ET [34] and pre-PMF [35]. JAK inhibitors, such as RUX or fedratinib, are approved for the treatment of myelofibrosis and may be considered in certain cases of pre-PMF, especially in patients with splenomegaly and symptoms. Participation in clinical trials may be an option for patients with pre-PMF, particularly when standard treatments are not effective or suitable. The treatment strategy for pre-PMF is often guided by risk stratification. For intermediate-1 and intermediate-2 risk patients, treatment is typically based on the presence and severity of symptoms or the need to prevent thromboembolic complications. High-risk patients may require more intensive management due to a poorer prognosis [36]. For patients with molecular high risk and signs of clonal evolution and disease progression, allogeneic hematopoietic cell transplantation should be considered.

### Relevant aspects of the panel discussion

- JAK inhibitors, such as ruxolitinib, have shown efficacy in addressing symptoms like splenomegaly in overt PMF but play a limited role in pre-PMF, where the primary therapeutic goals are preventing thrombotic events and achieving disease modification. The lack of evidence supporting their impact on disease progression highlights the need for alternative, targeted strategies. JAK-inhibitor treatment may be considered in patients with pre-PMF and symptomatic disease: Study data on ruxolitinib (RUX) [37–40] and prospective real-world data from the ERNEST study [41] both indicate an overall survival (OS) benefit from RUX therapy in overt PMF. Patients who are treated early with RUX may benefit more from the treatment [31]. RAS/CBL mutations may predict resistance to JAK inhibitor treatment [42]. Spleens of patients with low fibrosis grades respond better to early RUX treatment [31].
- It is generally known from clinical practice that a minimum daily dose of 30 mg RUX may be required to achieve a relevant spleen response. In cases with RAS/CBL1 or ASXL1 mutations, or in patients with a palpable spleen size greater than 10 cm below the costal margin, the spleen response was significantly impaired. OS is significantly worse in patients with RAS/CBL1 mutations during JAK inhibitor treatment (30 vs. 91 months). OS correlates with spleen response and with the maintenance of spleen response. The benefit of early RUX therapy is comparable in patients with low fibrosis grades to those with high fibrosis grades; both groups benefit more when therapy begins early [31]. The following factors worsen the success of therapy with JAK1/2 inhibitors regarding time to treatment failure and OS: transfusion dependence before starting JAK-inhibitor therapy, high DIPSS, and ASXL1 or EZH2 mutations [43].
- Formally, second generation JAK-inhibitors such as fedratinib, pacritinib and momelotinib have not been tested in defined cohorts of pre-PMF. However, clinical trials as well as retrospective cohorts, variably included patients in pre-fibrotic stages of myelofibrosis due to the clinical heterogeneity described above. In a retrospective comparison of clinical trial patients treated with momelotinib, RUX, fedratinib, or BMS-911,543, fedratinib showed superior spleen response rates, while momelotinib excelled in anemia response compared to RUX. Formally, fedratinib is approved for the treatment of disease-related splenomegaly or symptoms in adult patients with primary myelofibrosis or secondary myelofibrosis who are JAK-inhibitor naive or have been treated with RUX. Momelotinib is approved for the treatment of disease-related splenomegaly or symptoms in adult patients with moderate to severe anemia with primary or secondary myelofibrosis either treatment naive or who have been treated with RUX. Both definitions can (in principle) apply for patients in the pre-fibrotic phase of myelofibrosis.
- However, all previous studies have the limitation that they are retrospective studies, and there are no data available from randomized trials with “pre-PMF only” cohorts. The early-stage patients are typically younger, while the late-stage patients are usually older. Younger patients are often diagnosed early (and are in a less advanced stage of the disease) and frequently have many symptoms, whereas older patients are diagnosed later, perhaps because they had fewer symptoms (and are already in a more advanced stage). In JAK inhibitor studies, especially with long treatment durations, some severe side effects, such as skin cancers or complications of severe immunosuppression, can be observed. Even though overall, there were few side effects, the side effects of long-term treatment need to be critically examined, particularly in young patients. Patients with pre-PMF should therefore only be treated with JAK-inhibitors with a clear indication such as splenomegaly or symptoms, as long as there is no evidence that early treatment prevents progression. The ReThink study could have answered this question. Formally, the approvals are based on the COMFORT I and II studies when pre-PMF not (yet) defined by WHO classifications.
- Peg-IFN alfa 2a and 2b have emerged as promising agents in pre-PMF and MPNs, offering benefits in disease modification by reducing JAK2V617F allele burden and improving progression-free survival. Data from Silver et al. and the Mayo Clinic support its efficacy in mitigating thrombotic risk and delaying disease progression, particularly in younger, molecularly high-risk patients [23, 25, 33, 35, 44, 45]. Ropeginterferon-alpha is a potential and veritable treatment option in pre-PMF, which may be particularly beneficial in JAK2-mutated disease [46]. This is supported by recently published data [47]. Trials investigating Ropeginterferon-alpha in early/lower risk MF are currently ongoing [35].

## Data Availability

No datasets were generated or analysed during the current study.

## References

[1] Arber DA, Orazi A, Hasserjian RP, Borowitz MJ, Calvo KR, Kvasnicka HM, Wang SA, Bagg A, Barbui T, Branford S, Bueso-Ramos CE, Cortes J, Dal Cin P, DiNardo CD, Dombret H, Duncavage EJ, Ebert BL, Estey E, Facchetti F, Foucar K, Gangat N, Gianelli U, Godley LA, Goekbuget N, Gotlib JR, Hellstrom-Lindberg E, Hobbs G, Hoffman R, Jabbour EJ, Kiladjian JJ, Larson RA, Le Beau MM, Loh ML, Lowenberg B, Macintyre EA, Malcovati L, Mullighan CG, Niemeyer CM, Odenike O, Ogawa S, Orfao A, Papaemmanuil E, Passamonti F, Porkka K, Pui CH, Radich JP, Reiter A, Rozman M, Rudelius M, Savona MR, Schiffer C, Schmitt-Graeff A, Shimamura A, Sierra J, Stock W, Stone RM, Tallman MS, Thiele J, Tien HF, Tzankov A, Vannucchi AM, Vyas P, Wei AH, Weinberg OK, Wierzbowska A, Cazzola M, Dohner H, Tefferi A (2022) International Consensus Classification of Myeloid Neoplasms and Acute Leukemia: Integrating Morphological, Clinical, and Genomic Data. Blood. 10.1182/blood.2022015850

[2] Khoury JD, Solary E, Abla O, Akkari Y, Alaggio R, Apperley JF, Bejar R, Berti E, Busque L, Chan JKC, Chen W, Chen X, Chng WJ, Choi JK, Colmenero I, Coupland SE, Cross NCP, De Jong D, Elghetany MT, Takahashi E, Emile JF, Ferry J, Fogelstrand L, Fontenay M, Germing U, Gujral S, Haferlach T, Harrison C, Hodge JC, Hu S, Jansen JH, Kanagal-Shamanna R, Kantarjian HM, Kratz CP, Li XQ, Lim MS, Loeb K, Loghavi S, Marcogliese A, Meshinchi S, Michaels P, Naresh KN, Natkunam Y, Nejati R, Ott G, Padron E, Patel KP, Patkar N, Picarsic J, Platzbecker U, Roberts I, Schuh A, Sewell W, Siebert R, Tembhare P, Tyner J, Verstovsek S, Wang W, Wood B, Xiao W, Yeung C, Hochhaus A (2022) The 5th edition of the World Health Organization Classification of Haematolymphoid Tumours: Myeloid and Histiocytic/Dendritic Neoplasms. Leukemia. 36(7):1703–1719. 10.1038/s41375-022-01613-1 10.1038/s41375-022-01613-1 PMC9252913 35732831

[3] Schmidt A, Bernhardt C, Burkle D, Fries S, Hannig CV, Jentsch-Ullrich K, Josting A, Kreher S, Reiser M, Steinmetz HT, Tesch H, Terner S, Schulte A, Crodel CC, Palandri F, Heidel FH (2023) Diagnosis and treatment of MPN in real life: exploratory and retrospective chart review including 960 MPN patients diagnosed with ET or MF in Germany. J Cancer Res Clin Oncol 149(10):7197–7206. 10.1007/s00432-023-04669-3 10.1007/s00432-023-04669-3 PMC10374473 36884118

[4] Barbui T, Finazzi G, Vannucchi AM, De Stefano V (2018) Targeting myeloid cells to prevent recurrent stroke in general population: the lesson of hydroxyurea in myeloproliferative neoplasms. Blood Cancer J 8(11):103. 10.1038/s41408-018-0143-y 10.1038/s41408-018-0143-y PMC6221886 30405115

[5] Lin Y, Chen Q, Chen T (2024) Recent advancements in machine learning for bone marrow cell morphology analysis. Front Med (Lausanne) 11:p1402768. 10.3389/fmed.2024.1402768 10.3389/fmed.2024.1402768 PMC11211563 38947236

[6] Tayebi RM, Mu Y, Dehkharghanian T, Ross C, Sur M, Foley R, Tizhoosh HR, Campbell CJV (2022) Automated bone marrow cytology using deep learning to generate a histogram of cell types. Commun Med (Lond) 2:45. 10.1038/s43856-022-00107-6 10.1038/s43856-022-00107-6 PMC9053230 35603269

[7] van Eekelen L, Litjens G, Hebeda KM (2024) Artificial Intelligence in bone marrow histological Diagnostics: potential applications and challenges. Pathobiology 91(1):8–17. 10.1159/000529701 36791682 10.1159/000529701 PMC10937040

[8] Arber DA, Orazi A, Hasserjian R, Thiele J, Borowitz MJ, Le Beau MM, Bloomfield CD, Cazzola M, Vardiman JW (2016) The 2016 revision to the World Health Organization classification of myeloid neoplasms and acute leukemia. Blood. 127(20):2391–405. 10.1182/blood-2016-03-643544 10.1182/blood-2016-03-643544 27069254

[9] Harrison C, Ross DM, Fogliatto LM, Foltz L, Busque L, Xiao Z, Heidel FH, Koehler M, Palumbo GA, Breccia M, Komatsu N, Kirito K, Cirici BX, Martinez-Lopez J, Rovo A, Petruk C, Bobirca C, Mirams L, McMillan A, Harper G, Kiladijan JJ (2024) Patient and physician perceptions regarding treatment expectations and symptomatology in polycythemia vera: Insights from the Landmark 2.0 Global Health Survey. Hemasphere. In revision 10.1002/hem3.70106 PMC11931442 40130070

[10] Manz K, Heidel FH, Koschmieder S, Schlag R, Lipke J, Stegelmann F, Griesshammer M, Klausmann M, Crodel CC, Hochhaus A, Schulz H, Göthert JR, Al-Ali HK, Becker H, Reiter A, Beutel G, Kricheldorf K, Brummendorf TH, Hoffmann W, Döhner K, Isfort S (2024) Comparison of recognition of symptom burden in MPN between patient- and physician-reported assessment– an intraindividual analysis by the German study group for MPN (GSG-MPN). Leukemia. In press 10.1038/s41375-025-02524-7 PMC11976279 40000843

[11] Grinfeld J, Nangalia J, Baxter EJ, Wedge DC, Angelopoulos N, Cantrill R, Godfrey AL, Papaemmanuil E, Gundem G, MacLean C, Cook J, O’Neil L, O’Meara S, Teague JW, Butler AP, Massie CE, Williams N, Nice FL, Andersen CL, Hasselbalch HC, Guglielmelli P, McMullin MF, Vannucchi AM, Harrison CN, Gerstung M, Green AR, Campbell PJ (2018) Classification and Personalized Prognosis in Myeloproliferative Neoplasms. N Engl J Med. 379(15):1416–1430. 10.1056/NEJMoa1716614 10.1056/NEJMoa1716614 PMC7030948 30304655

[12] Guglielmelli P, Lasho TL, Rotunno G, Mudireddy M, Mannarelli C, Nicolosi M, Pacilli A, Pardanani A, Rumi E, Rosti V, Hanson CA, Mannelli F, Ketterling RP, Gangat N, Rambaldi A, Passamonti F, Barosi G, Barbui T, Cazzola M, Vannucchi AM, Tefferi A (2018) MIPSS70: mutation-enhanced international prognostic score system for transplantation-age patients with primary myelofibrosis. J Clin Oncol 36(4):310–318. 10.1200/JCO.2017.76.4886 29226763 10.1200/JCO.2017.76.4886

[13] Jeryczynski G, Thiele J, Gisslinger B, Wolfler A, Schalling M, Gleiss A, Burgstaller S, Buxhofer-Ausch V, Sliwa T, Schlogl E, Geissler K, Krauth MT, Nader A, Vesely M, Simonitsch-Klupp I, Mullauer L, Beham-Schmid C, Gisslinger H (2017) Pre-fibrotic/early primary myelofibrosis vs. WHO-defined essential thrombocythemia: The impact of minor clinical diagnostic criteria on the outcome of the disease. Am J Hematol. 92(9):885–891. 10.1002/ajh.24788 10.1002/ajh.24788 28543356

[14] Gur HD, Loghavi S, Garcia-Manero G, Routbort M, Kanagal-Shamanna R, Quesada A, Khogeer H, Pierce S, Medeiros LJ, Kantarjian H, Khoury JD (2018) Chronic Myelomonocytic Leukemia With Fibrosis Is a Distinct Disease Subset With Myeloproliferative Features and Frequent JAK2 p.V617F Mutations. Am J Surg Pathol. 42(6):799–806. 10.1097/PAS.0000000000001058 10.1097/PAS.0000000000001058 29596070

[15] Hu Z, Ramos CEB, Medeiros LJ, Zhao C, Yin CC, Li S, Hu S, Wang W, Thakral B, Xu J, Verstovsek S, Lin P (2019) Utility of JAK2 V617F allelic burden in distinguishing chronic myelomonocytic Leukemia from Primary myelofibrosis with monocytosis. Hum Pathol. 85:290–298. 10.1016/j.humpath.2018.10.026 10.1016/j.humpath.2018.10.026 30447300

[16] Pophali P, Lasho TL, Finke C, Horna P, Ketterling RP, Gangat N, Begna K, Mangaonkar AA, Pardanani A, Tefferi A, Patnaik MM (2018) Clinical Correlates, Prognostic Impact and Survival Outcomes in Chronic Myelomonocytic Leukemia Patients with Myeloproliferative Neoplasm Associated-Driver Mutations. Blood (ASH Annual Meeting Abstracts). 132:3100. 10.1182/blood-2018-99-117335

[17] Vachhani P, Loghavi S, Bose P (2024) SOHO State of the Art Updates and Next Questions| Diagnosis, Outcomes, and Management of Prefibrotic Myelofibrosis. Clin Lymphoma Myeloma Leuk. 24(7):413–426. 10.1016/j.clml.2024.01.009 10.1016/j.clml.2024.01.009 38341324

[18] Carobbio A, Guglielmelli P, Rumi E, Cavalloni C, De Stefano V, Betti S, Rambaldi A, Finazzi MC, Thiele J, Vannucchi AM, Tefferi A, Barbui T (2020) A multistate model of survival prediction and event monitoring in prefibrotic myelofibrosis. Blood Cancer J. 10(10):100. 10.1038/s41408-020-00368-1 10.1038/s41408-020-00368-1 PMC7566465 33056979

[19] Barbui T, Barosi G, Birgegard G, Cervantes F, Finazzi G, Griesshammer M, Harrison C, Hasselbalch HC, Hehlmann R, Hoffman R, Kiladjian JJ, Kroger N, Mesa R, McMullin MF, Pardanani A, Passamonti F, Vannucchi AM, Reiter A, Silver RT, Verstovsek S, Tefferi A, European L (2011) Philadelphia-negative classical myeloproliferative neoplasms: critical concepts and management recommendations from European LeukemiaNet. J Clin Oncol. 29(6):761–70. 10.1200/JCO.2010.31.8436 10.1200/JCO.2010.31.8436 PMC4979120 21205761

[20] Guglielmelli P, Carobbio A, Rumi E, De Stefano V, Mannelli L, Mannelli F, Rotunno G, Coltro G, Betti S, Cavalloni C, Finazzi MC, Thiele J, Cazzola M, Vannucchi AM, Barbui T (2020) Validation of the IPSET score for thrombosis in patients with prefibrotic myelofibrosis. Blood Cancer J. 10(2):21. 10.1038/s41408-020-0289-2 10.1038/s41408-020-0289-2 PMC7042364 32098944

[21] Perner F, Perner C, Ernst T, Heidel FH (2019) Roles of JAK2 in Aging, Inflammation, Hematopoiesis and Malignant Transformation. Cells. 8(8). 10.3390/cells8080854 10.3390/cells8080854 PMC6721738 31398915

[22] Guglielmelli P, Biamonte F, Rotunno G, Artusi V, Artuso L, Bernardis I, Tenedini E, Pieri L, Paoli C, Mannarelli C, Fjerza R, Rumi E, Stalbovskaya V, Squires M, Cazzola M, Manfredini R, Harrison C, Tagliafico E, Vannucchi AM, Investigators C-I (2013) and I. Associazione Italiana per la Ricerca sul Cancro Gruppo Italiano Malattie Mieloproliferative, 2014. Impact of mutational status on outcomes in myelofibrosis patients treated with ruxolitinib in the COMFORT-II study. Blood. 123(14):2157–60. 10.1182/blood--11-536557 10.1182/blood-2013-11-536557 24458439

[23] Tefferi A, Guglielmelli P, Larson DR, Finke C, Wassie EA, Pieri L, Gangat N, Fjerza R, Belachew AA, Lasho TL, Ketterling RP, Hanson CA, Rambaldi A, Finazzi G, Thiele J, Barbui T, Pardanani A, Vannucchi AM (2014) Long-term survival and blast transformation in molecularly annotated essential thrombocythemia, polycythemia vera, and myelofibrosis. Blood. 124(16):2507–13. 10.1182/blood-2014-05-579136 10.1182/blood-2014-05-579136 PMC4199952 25037629

[24] Vannucchi AM, Lasho TL, Guglielmelli P, Biamonte F, Pardanani A, Pereira A, Finke C, Score J, Gangat N, Mannarelli C, Ketterling RP, Rotunno G, Knudson RA, Susini MC, Laborde RR, Spolverini A, Pancrazzi A, Pieri L, Manfredini R, Tagliafico E, Zini R, Jones A, Zoi K, Reiter A, Duncombe A, Pietra D, Rumi E, Cervantes F, Barosi G, Cazzola M, Cross NC, Tefferi A (2013) Mutations and prognosis in primary myelofibrosis. Leukemia. 27(9):1861–9. 10.1038/leu.2013.119 10.1038/leu.2013.119 23619563

[25] Tefferi A (2023) Primary myelofibrosis: 2023 update on diagnosis, risk-stratification, and management. Am J Hematol. 98(5):801–821. 10.1002/ajh.26857 10.1002/ajh.26857 36680511

[26] Guglielmelli P, Pacilli A, Rotunno G, Rumi E, Rosti V, Delaini F, Maffioli M, Fanelli T, Pancrazzi A, Pietra D, Salmoiraghi S, Mannarelli C, Franci A, Paoli C, Rambaldi A, Passamonti F, Barosi G, Barbui T, Cazzola M, Vannucchi AM, Group A (2017) Presentation and outcome of patients with 2016 WHO diagnosis of prefibrotic and overt primary myelofibrosis. Blood. 129(24):3227–3236. 10.1182/blood-2017-01-761999 10.1182/blood-2017-01-761999 28351937

[27] Palandri F, Sabattini E, Maffioli M (2019) Treating early-stage myelofibrosis. Ann Hematol. 98(2):241–253. 10.1007/s00277-018-3526-z 10.1007/s00277-018-3526-z 30343328

[28] Heidel FH, Platzbecker U (2021) The Genomic Landscape of Myeloid Malignancies: Options for Pan-myeloid Therapies?. Hemasphere. 5(3):e537. 10.1097/HS9.0000000000000537 10.1097/HS9.0000000000000537 PMC7886402 33604515

[29] Kuo MC, Chuang WY, Chang H, Lin TH, Wu JH, Lin TL, Ou CW, Hung YS, Huang TY, Huang YJ, Wang PN, Shih LY (2023) Comparison of Clinical and Molecular Features Between Patients With Essential Thrombocythemia and Early/Prefibrotic Primary Myelofibrosis Presenting With Thrombocytosis in Taiwan. Am J Clin Pathol. 159(5):474–483. 10.1093/ajcp/aqac173 10.1093/ajcp/aqac173 36857745

[30] Coltro G, Mannelli F, Loscocco GG, Mannarelli C, Rotunno G, Maccari C, Pancani F, Atanasio A, Vannucchi AM, Guglielmelli P (2022) Differential prognostic impact of cytopenic phenotype in prefibrotic vs overt primary myelofibrosis. Blood Cancer J. 12(8):116. 10.1038/s41408-022-00713-6 10.1038/s41408-022-00713-6 PMC9374751 35961958

[31] Palandri F, Al-Ali HK, Guglielmelli P, Zuurman MW, Sarkar R, Gupta V (2023) Benefit of Early Ruxolitinib Initiation Regardless of Fibrosis Grade in Patients with Primary Myelofibrosis: A Post Hoc Analysis of the Single-Arm Phase 3b JUMP Study. Cancers (Basel). 15(10). 10.3390/cancers15102859 10.3390/cancers15102859 PMC10216208 37345196

[32] Petrides PE, Baerlocher GM, Döhner K, Gisslinger H, Griesshammer M, Koschmieder S, Schwaab J, Lengfelder E (2023) Essentielle (oder primäre) Thrombozythämie (ET). Onkopedia - Leitlinien der DGHO

[33] Abu-Zeinah G, Krichevsky S, Cruz T, Hoberman G, Jaber D, Savage N, Sosner C, Ritchie EK, Scandura JM, Silver RT (2021) Interferon-alpha for treating polycythemia vera yields improved myelofibrosis-free and overall survival. Leukemia. 35(9):2592–2601. 10.1038/s41375-021-01183-8 10.1038/s41375-021-01183-8 PMC8410882 33654206

[34] Kiladjian JJ, Marin FF, Al-Ali HK, Alvarez-Larran A, Beggiato E, Bieniaszewska M, Breccia M, Buxhofer-Ausch V, Cerna O, Crisan AM, Danaila CD, De Stefano V, Dohner K, Empson V, Gora-Tybor J, Griesshammer M, Grosicki S, Guglielmelli P, Garcia-Gutierrez V, Heidel FH, Illes A, Tomuleasa C, James C, Koschmieder S, Krauth MT, Krejcy K, Lazaroiu MC, Mayer J, Nagy ZG, Nicolini FE, Palandri F, Pappa V, Reiter AJ, Sacha T, Schlager S, Schmidt S, Terpos E, Unger M, Wolfler A, Cirici BX, Klade C (2024) *ROP-ET: a prospective phase III trial investigating the efficacy and safety of ropeginterferon alfa-2b in essential thrombocythemia patients with limited treatment options.* Ann Hematol. 103(7):2299–2310. 10.1007/s00277-024-05665-4 10.1007/s00277-024-05665-4 PMC11224110 38438627

[35] Abu-Zeinah G, Qin A, Gill H, Komatsu N, Mascarenhas J, Shih WJ, Zagrijtschuk O, Sato T, Shimoda K, Silver RT, Mesa R (2024) A randomized, double-blind, placebo-controlled phase 3 study to assess efficacy and safety of ropeginterferon alfa-2b in patients with early/lower-risk primary myelofibrosis. Ann Hematol. 10.1007/s00277-024-05912-8 39145781 10.1007/s00277-024-05912-8 PMC11358163

[36] Finazzi G, Vannucchi AM, Barbui T (2018) *Prefibrotic myelofibrosis: treatment algorithm 2018.* Blood Cancer J. 8(11):104. 10.1038/s41408-018-0142-z 10.1038/s41408-018-0142-z PMC6221891 30405096

[37] Harrison C, Kiladjian JJ, Al-Ali HK, Gisslinger H, Waltzman R, Stalbovskaya V, McQuitty M, Hunter DS, Levy R, Knoops L, Cervantes F, Vannucchi AM, Barbui T, Barosi G (2012) JAK inhibition with ruxolitinib versus best available therapy for myelofibrosis. N Engl J Med. 366(9):787–98. 10.1056/NEJMoa1110556 10.1056/NEJMoa1110556 22375970

[38] Harrison CN, Vannucchi AM, Kiladjian JJ, Al-Ali HK, Gisslinger H, Knoops L, Cervantes F, Jones MM, Sun K, McQuitty M, Stalbovskaya V, Gopalakrishna P, Barbui T (2017) Long-term findings from COMFORT-II, a phase 3 study of ruxolitinib vs best available therapy for myelofibrosis. Leukemia. 31(3):775. 10.1038/leu.2016.323 10.1038/leu.2016.323 PMC7609269 28248313

[39] Verstovsek S, Gotlib J, Mesa RA, Vannucchi AM, Kiladjian JJ, Cervantes F, Harrison CN, Paquette R, Sun W, Naim A, Langmuir P, Dong T, Gopalakrishna P, Gupta V (2017) Long-term survival in patients treated with ruxolitinib for myelofibrosis: COMFORT-I and -II pooled analyses. J Hematol Oncol. 10(1):156. 10.1186/s13045-017-0527-7 10.1186/s13045-017-0527-7 PMC5622445 28962635

[40] Verstovsek S, Mesa RA, Gotlib J, Levy RS, Gupta V, DiPersio JF, Catalano JV, Deininger M, Miller C, Silver RT, Talpaz M, Winton EF, Harvey JH Jr., Arcasoy MO, Hexner E, Lyons RM, Paquette R, Raza A, Vaddi K, Erickson-Viitanen S, Koumenis IL, Sun W, Sandor V, Kantarjian HM (2012) A double-blind, placebo-controlled trial of ruxolitinib for myelofibrosis. N Engl J Med. 366(9):799–807. 10.1056/NEJMoa1110557 10.1056/NEJMoa1110557 PMC4822164 22375971

[41] Guglielmelli P, Ghirardi A, Carobbio A, Masciulli A, Maccari C, Mora B, Rumi E, Triguero A, Finazzi MC, Pettersson H, Paoli C, Mannelli F, Vanni D, Rambaldi A, Passamonti F, Alvarez-Larran A, Andreasson B, Vannucchi AM, Barbui T (2022) Impact of ruxolitinib on survival of patients with myelofibrosis in the real world: update of the ERNEST Study. Blood Adv. 6(2):373–375. 10.1182/bloodadvances.2021006006 10.1182/bloodadvances.2021006006 PMC8791590 34753179

[42] Coltro G, Rotunno G, Mannelli L, Mannarelli C, Fiaccabrino S, Romagnoli S, Bartalucci N, Ravenda E, Gelli E, Sant’Antonio E, Patnaik MM, Tefferi A, Vannucchi AM, Guglielmelli P (2020) RAS/CBL mutations predict resistance to JAK inhibitors in myelofibrosis and are associated with poor prognostic features. Blood Adv. 4(15):3677–3687. 10.1182/bloodadvances.2020002175 10.1182/bloodadvances.2020002175 PMC7422130 32777067

[43] Spiegel JY, McNamara C, Kennedy JA, Panzarella T, Arruda A, Stockley T, Sukhai M, Thomas M, Bartoszko J, Ho J, Siddiq N, Maze D, Schimmer A, Schuh A, Sibai H, Yee K, Claudio J, Devlin R, Minden MD, Kamel-Reid S, Gupta V (2017) Impact of genomic alterations on outcomes in myelofibrosis patients undergoing JAK1/2 inhibitor therapy. Blood Adv. 1(20):1729–1738. 10.1182/bloodadvances.2017009530 10.1182/bloodadvances.2017009530 PMC5728340 29296819

[44] Silver RT, Vandris K, Goldman JJ (2011) Recombinant interferon-alpha may retard progression of early primary myelofibrosis: a preliminary report. Blood. 117(24):6669–72. 10.1182/blood-2010-11-320069 10.1182/blood-2010-11-320069 21518929

[45] Tefferi A (2013) *Polycythemia vera and essential thrombocythemia: 2013 update on diagnosis, risk-stratification, and management.* Am J Hematol. 88(6): pp. 507–16. 10.1002/ajh.23417 10.1002/ajh.23417 23695894

[46] Czech J, Cordua S, Weinbergerova B, Baumeister J, Crepcia A, Han L, Maie T, Costa IG, Denecke B, Maurer A, Schubert C, Feldberg K, Gezer D, Brummendorf TH, Muller-Newen G, Mayer J, Racil Z, Kubesova B, Knudsen T, Sorensen AL, Holmstrom M, Kjaer L, Skov V, Larsen TS, Hasselbalch HC, Chatain N, Koschmieder S (2019) JAK2V617F but not CALR mutations confer increased molecular responses to interferon-alpha via JAK1/STAT1 activation. Leukemia. 33(4):995–1010. 10.1038/s41375-018-0295-6 10.1038/s41375-018-0295-6 30470838

[47] Gill H, Au L, Leung GMK, Tsai D, Yim R, Chin L, Li V, Lee P, Sin ACF, Hou HA, Chen C-C, Kwong YL (2023) Ropeginterferon Alfa-2b for Pre-Fibrotic Primary Myelofibrosis and DIPSS Low/Intermediate-1 Risk Myelofibrosis: Durable Responses and Evidence of Disease Modification. Blood (ASH Annual Meeting Abstracts). 142:4562

